# The Impact of Viral Load on the Severity and Outcome Among Patients With COVID-19: A Cross-Sectional Study

**DOI:** 10.7759/cureus.64137

**Published:** 2024-07-09

**Authors:** Fatma M Shire, Shatha Sharbatti, Firas AlNajjar, Lara Sulaiman Abumuaileq, Rand Abuelkher, Hebah Sabri, Aasiya Beevi, Alia Alqahtani, Rami Beshtawy

**Affiliations:** 1 Emergency Department, Rashid Hospital, Dubai Academic Health Corporation, Dubai, ARE; 2 Community Medicine Department, Gulf Medical University, Ajman, ARE; 3 Medical Education Department, Dubai Medical College for Girls, Dubai, ARE

**Keywords:** cycle threshold (ct) value, viral load, sars-cov-2, severity, covid-19

## Abstract

Objectives

This study aimed to assess the relationship between illness severity and mortality among COVID-19 patients along with the cycle threshold (Ct) value measured by viral load.

Methods

A cross-sectional study was conducted based on records of the emergency room at Rashid Hospital located in Dubai, United Arab Emirates. This research was carried out on all of the appropriate records of patients who were hospitalized at Rashid Hospital in Dubai between May 2020 and January 2021. Clinical and laboratory data were used as severity indicators, and in-hospital death was designated as the outcome.

Results

A total of 1,633 cases were included in the analysis. The percentage of deceased patients was higher in patients with a low Ct value (11.6%) than in patients with a high Ct value (6.9%) (p-value = 0.003). Logistic analysis revealed a statistically significant correlation (OR=2.046; p-value=0.002) between mortality and viral load, as measured by the Ct value. Patients with low Ct values and aberrant laboratory findings had a higher frequency of respiratory problems and required oxygen therapy, according to clinical and laboratory markers.

Conclusions

A correlation was found between viral load and mortality. Advanced age, history of chronic disease, and abnormal clinical and laboratory findings were all independently linked to a greater mortality rate in COVID-19 patients, indicating that they might be utilized as predictive and prognostic factors along with the viral load.

## Introduction

The coronavirus disease 2019 (COVID-19) was revealed in Wuhan, China, and has claimed many lives globally. Many experienced pneumonia-like symptoms, but doctors could not figure out what was causing it [1].

The two main methods for detecting the coronavirus that causes severe acute respiratory syndrome (SARS-CoV-2) are reverse transcription polymerase chain reaction (RT-PCR) and nucleic acid extraction. An antigen test might be utilized as the main examination if the standard test was not obtainable or was too costly. However, the antigen test's sensitivity is lower than the diagnostic tests, therefore additional standardized testing might be necessary if the antigen test results are negative [2].

Healthcare professionals can detect the presence or absence of SARS-CoV-2 based on the RT-PCR test. Furthermore, it may assess quantitative cycle threshold (Ct) values that are inversely linked to viral load, although this information is not clinically reported.

The amount of viral ribonucleic acid (RNA), expressed as infectious particles per milliliter, present in a given volume is defined in some literature reviews as the "viral load." An alternative way to write this would be "Log 10 copies per milliliter” [3].

The number of amplification cycles required to detect a target gene at a level beyond its threshold detection level is expressed mathematically as the Ct value. When Ct readings are below normal, the viral load increases. As a result, the Ct value may be the starting point for developing an indirect technique to quantify the quantity of viral RNA copies in a COVID-19 sample swab [4].

Researchers have a conflict regarding whether viral RNA levels in respiratory samples correlate with the severity or mortality of an infection or not. The relationship between Ct levels and the course of sickness is supported by the literature, as 286 COVID-19 PCR-positive cases were investigated by Tsukagoshi et al., in 2021 in Gunma Prefecture, Japan. It was inferred that the virus load in the deceased cases was found to be much larger than in the symptomatic or asymptomatic survived patients [5].

On the other hand, numerous investigations have not discovered a difference in viral loads, as shown by Ct values, between symptomatic and asymptomatic patients, regardless of the severity of the illness. A community treatment center in Korea housed 303 COVID-19 patients, both symptomatic and asymptomatic, in isolation as of March 2020. On these patients, Lee et al. conducted a retrospective cohort analysis and found no appreciable difference in viral loads between those who had symptoms and those who did not [6].

Because of the inconsistent findings in different research, the relationship of viral load with hospitalization risk in both inpatients and outpatients has remained unknown and is still under investigation [5,6].

Currently, a nasopharyngeal swab is obtained and COVID-19 RNA is detected utilizing an RT-PCR as part of the standard diagnostic test for COVID-19 in the United Arab Emirates (UAE). Thus far, the RT-PCR test notifies medical professionals about the virus’ presence or absence without determining the amount of viral load [7].

The study question that was put out was; “Would a unique SARS-CoV-2 viral load from the initial nasopharyngeal swab sample function as a helpful therapeutic tool for assessing the severity and mortality of patients in light of the previously provided information?” Therefore, the purpose of this study was to look into the connection between viral loads and the severity of the sickness. The study's goal was to detect severe cases early on and treat them vigorously to enhance outcomes and reduce complications. The primary objectives of this study were to evaluate the relationship between viral load levels and the clinical course of patients diagnosed with COVID-19, as well as the relationship between viral load levels and the likelihood of death.

This type of research was important because it may be possible to predict a patient's course and stratify the likelihood of oxygen demand or intubation based on the viral load of the virus. Additionally, to track the correlation between the illness's severity and the viral load, as well as the virus' potential to propagate in the future. With this information, the government might then be better equipped to enforce quarantine and contact-tracking public health regulations.

## Materials and methods

Study design and setting

This was a record-based retrospective, single-center, cross-sectional study carried out in Rashid Hospital, Dubai, from May 2020 to January 2021. Rashid Hospital is a tertiary hospital that covers a wide range of cases and is regarded as one of the leading institutions in the treatment of COVID-19 patients.

Inclusion and exclusion criteria

The study included all patients who tested positive for SARS-CoV-2 infection and were ≥18 years of age, treated at Rashid Hospital during the study period, regardless of their gender or nationality, as long as Ct values were available. However, patients under the age of 18 years, pregnant women, tests for which Ct values were unavailable, and records with incomplete medical data were excluded.

Study instrument and validation procedure

The recorded data was retrieved using a form. The form contained details about the patient's demographics, medical history, both past and present, clinical findings, admission vital signs, laboratory results, and radiological studies - all of which were obtained directly from the patient's electronic medical data. Furthermore, information about the hospital stay, results, and problems was obtained from the data. The results of the initial nasopharyngeal swab test were used to compute the Ct values in cooperation with the pathology and genetics department.

Every hospitalized patient in the emergency room underwent a nasopharyngeal test. The existence of infection was confirmed by an RT-PCR study using a SARS-CoV-2 nucleic acid detection kit, and the viral load was determined by an analysis of the positive results for both the N2 and E genes using the SARS-CoV-2 Cepheid -Xpert Omni kit (Cepheid, Sunnyvale, California, USA). The viral load was ascertained using the RT-PCR assay result following the manufacturer's instructions. A positive result was defined as having two genes detected by a Ct value of less than 40, and a negative result was defined as having no genes detected by a Ct value of more than 40. Plotting Ct data onto the standard curve created using the standard product allowed for the calculation of viral load [8].

The categories used in the study, which were stratified by the Ministry of Health to describe the severity of diseases, were asymptomatic upper respiratory tract infections (URTIs), mild to moderate (i.e., no oxygen requirement, no clinical evidence of pneumonia but with other symptoms, such as fever and diarrhea), severe (requiring oxygen therapy), and critical cases [9].

Ethics approval

The research and ethical committee of the Dubai Health Authority as well as the Institutional Review Board (IRB) of Gulf Medical University authorized the study with reference number IRB/COM/STD/43/Dec-2021. The Gulf Medical University IRB and the Dubai Scientific Research Ethics Committee waived the requirement for informed consent because the study was retrospective.

Data analysis

For statistical analysis, the Statistical Package for the Social Sciences (IBM SPSS Statistics for Windows, IBM Corp., Version 26.0, Armonk, NY) was used. There was the use of both descriptive and inferential statistics. Categorical factors like gender, CXR values, WHO regions, comorbidities, complaints, and outcomes were examined using the logistic regression models and the chi-square test. Relationships were found using basic univariate logistic regression, with a p-value of 0.05 being deemed significant. Significant variables from the univariate research were used in a multivariate analysis to find pertinent qualities.

## Results

The current analysis includes 1,633 patients, with an average patient age of 46.63 years (SD±15.04). Men made up the majority of patients in this study (67.5%), and the majority of patients' original regions of origin were the Mediterranean (46.4%) and Eastern Southeast Asia (34%). The majority of research participants had dyslipidemia, diabetes, and hypertension, indicating the prevalence of co-occurring diseases in the population (Table 1).

**Table 1:** The Socio-Demographic Characteristics and Comorbidities of Patients * Other regions (Africa, Americas, Europe)

Variable	All patients (N=1336)		
Demographic	Subcategory	Numbers (n)	Percentages (%)
Age (year)	45 years and younger	844	51.7
	46-64 years	565	34.6
	>65 year	224	13.7
Gender	Male	1102	67.5
	Female	531	32.5
Nationality (WHO Region)	Eastern Mediterranean	756	46.4
	Southeast Asia	554	34.0
	Western Pacific	230	14.1
	Other regions*	88	5.4
Clinical			
Hypertension	Yes	459	28.2
	No	1168	71.8
Diabetes	Yes	449	27.6
	No	1180	72.4
Cardiovascular disease	Yes	147	9.0
	No	1482	91
Obstructive lung disease	Yes	78	4.8
	No	1551	95.2
Neurological disease	Yes	67	4.1
	No	1562	95.9
Dyslipidemia	Yes	177	10.9
	No	1451	89.1

Approximately, 5.5% of cases were sent straight to the intensive care unit; the majority (74.8%) were admitted to the low-dependency care ward. Patients presenting with fever (76.8%), cough (63.5%), and dyspnea (45.3%) were the most common. The findings revealed that 63.2% of the patients had aberrant results on their chest X-rays, 5.8% required mechanical breathing, 16.5% had received oxygen through various treatment modalities, and 14.6% of the patients had low oxygen saturation.

**Figure 1:**
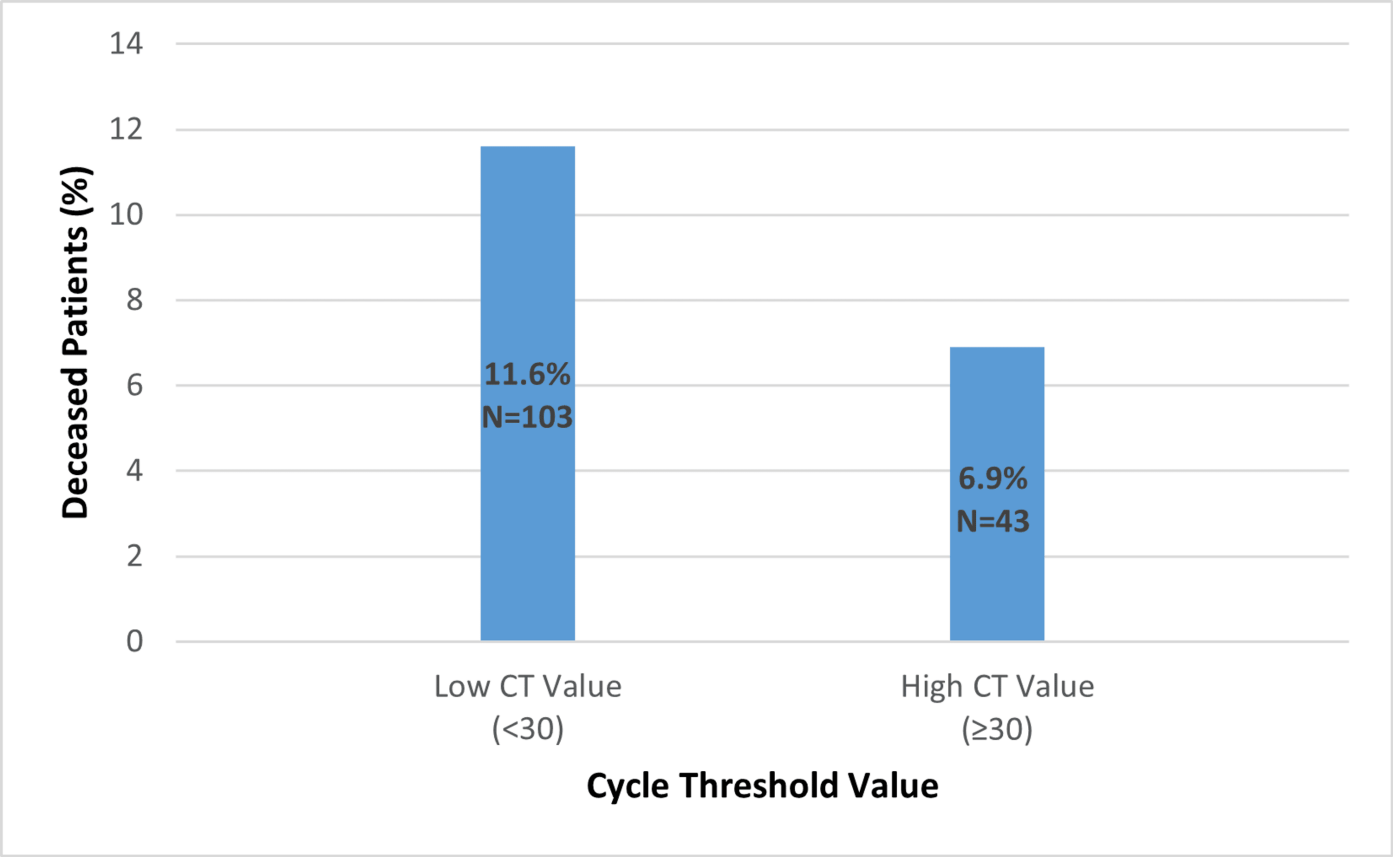
The Association Between the Cycle Threshold (Ct) Value With the Mortality p-value=0.003

Patients were distributed according to their Ct value. In general, patients had low Ct values (<30), suggesting a high viral load, and high Ct values (≥30), indicating a low viral load (41.3%). Moreover, a notable correlation between a low Ct value was observed, which indicated a high viral load, and the outcome of the patients treated at Rashid Hospital (p-value=0.003) (Figure 1).

Those aged >65 years had a larger proportion of deaths (4.4% vs. 11.3% vs. 24.1%, p-value=0.001) than young and middle-aged age. It was also greater among males (10.5% vs. 7.3%, p-value=0.047) compared to females, and among patients from Eastern Mediterranean region nations compared to those from other countries (10.8%, p-value=0.016).

**Figure 2:**
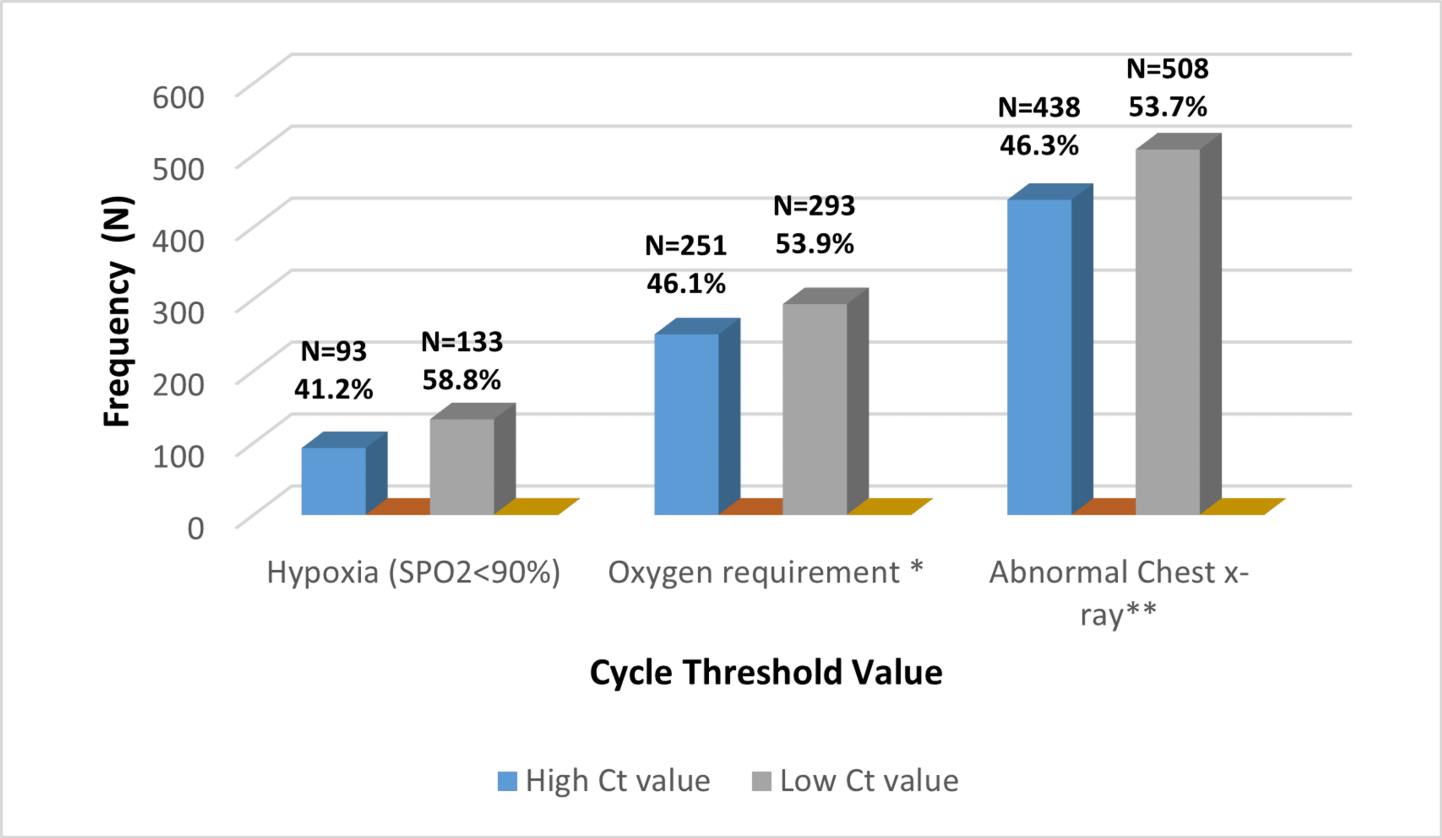
The Association Between Cycle Threshold Value With Clinical and Radiological Parameters * Oxygen requirements include a nasal cannula, non-rebreather mask (NRM), non-invasive ventilation (NIV), high-flow nasal cannula (HFNC), and intubation. ** Abnormal chest X-ray included consolidation, pleural effusion, lung mass, TB, and interstitial infiltrations.

Furthermore, it was demonstrated that the number of patients with hypoxia and who need oxygen was higher in those with low Ct values compared with the number of patients who had high Ct values, as well as the frequency of abnormal chest findings (Figure 2).

The proportion of deaths was significantly higher among patients who came with hypoxia (SPO2<90%) and required oxygen therapy compared to the other groups (25.1% vs. 6.8% p-value=0.001), (18.1% vs. 4.5% p-value=0.001), respectively. Additionally, the increased frequency of aberrant laboratory findings was reported particularly the proportion of lymphopenia among patients with low cycle thresholds. A slight elevation in the levels of C-reactive protein (CRP), serum ferritin, creatinine level, risk of cardiac injury and thromboembolic events, as well as high alanine aminotransferase (ALT) have existed among patients who had low Ct values (Table 2).

**Table 2:** The Association Between Cycle Threshold Value with Laboratory Parameters The bold text indicates statistically significant results. LDH: lactate dehydrogenase; CRP: C-reactive protein; ALT: alanine aminotransferase ^+ ^Cardiac injury: Positive troponin >14 ng/dL.

Variable	Subcategory	High Cycle Threshold Value		The Low Cycle Threshold Value		p-value
		Number	%	Number	%	
Low lymphocyte count	Yes	362	37.9	592	62.1	0.001
	No	263	46.9	298	53.1	
Elevated LDH	Yes	247	50.4	243	49.6	<0.001
	No	377	36.8	647	63.2	
Elevated CRP	Yes	447	45.7	532	54.3	<0.001
	No	166	32.7	341	67.3	
Elevated serum ferritin	Yes	388	47.4	430	52.6	<0.001
	No	236	34.1	456	65.9	
Cardiac injury^+^	Yes	321	47.2	359	52.8	<0.001
	No	304	36.5	530	63.5	
Thromboembolism	Yes	378	48.5	401	51.5	<0.001
	No	197	32.7	405	67.3	
High creatinine	Yes	69	42.9	92	57.1	<0.001
	No	555	41	798	59	
High ALT	Yes	243	47.9	264	52.1	<0.001
	No	382	37.9	626	62.1	

The logistic analysis demonstrated a statistically significant relation between viral load, as measured by the Ct value, and mortality (OR=2.046 p-value=0.002). These indicators include sociodemographic and comorbidities such as advanced age (OR=4.643; p-value=0.001), male gender (OR=1.720; p-value=0.034), obstructive lung disease (OR=2.817; p-value=0.008), neurological disease (OR=2.023; p-value=0.039), as well as abnormal clinical and laboratory findings such as hypoxia (OR=2.251; p-value=0.001), the need of oxygen therapy (OR=2.419; p-value=0.002), elevation of lactate dehydrogenase (LDH) enzyme (OR=2.262; p-value=0.001), and cardiac injury (OR=2.081; p-value=0.01) (Table 3).

**Table 3:** Logistic Analysis of Predictors for In-Hospital Mortality The bold text indicates the statistical significance of the odds ratio of variables. LDH: lactate dehydrogenase; ALT: alanine aminotransferase

Variable	Subcategory	Crude OR	CI 95%		P-value	Adjusted OR	CI 95%		P-value
Age	46-64 years	2.783	1.829	4.235	<0.001	2.102	1.229	3.595	0.007
	>60 years	6.920	4.413	10.850	<0.001	4.643	2.344	9.197	<0.001
Gender	Male	1.486	1.017	2.169	0.040	1.720	1.042	2.839	0.034
Low cycle threshold value (<30)		1.768	1.219	2.564	0.003	2.046	1.310	3.194	0.002
Hypoxia		4.580	3.198	6.558	<0.001	2.251	1.383	3.663	0.001
Oxygen therapy requirement		4.744	3.306	6.808	<0.001	2.419	1.379	4.243	0.002
Abnormal chest X-ray		2.124	1.436	3.139	<0.001	0.468	0.243	0.901	0.023
Elevated LDH		3.724	2.646	5.243	<0.001	2.626	1.495	4.615	0.001
Cardiac injury		2.047	1.459	2.870	<0.001	2.081	1.339	3.235	0.001
Elevated C-reactive protein		1.891	1.273	2.808	0.002	0.568	0.298	1.083	0.086
Obstructive lung disease		1.983	1.067	3.686	0.030	2.817	1.311	6.052	0.008
Hypertension		2.939	2.099	4.116	<0.001	1.434	0.878	2.342	0.150
Diabetes mellitus		1.928	1.371	2.712	<0.001	0.904	0.563	1.454	0.678
Dyslipidemia		1.763	1.120	2.776	0.014	0.798	0.422	1.509	0.488
Cardiovascular disease		2.011	1.253	3.226	0.004	0.663	0.342	1.285	0.223
Neurological disease		4.148	2.370	7.258	<0.001	2.203	1.041	4.665	0.039
Lymphopenia		1.686	1.159	2.453	0.006	1.229	0.753	2.005	0.409
Elevated ALT		1.402	0.999	1.968	0.051	1.051	0.666	1.658	0.832
Elevated ferritin		2.380	1.646	3.442	<0.001	1.353	0.769	2.38	0.294
Thromboembolism		2.611	1.750	3.895	<0.001	1.092	0.642	1.856	0.745
Renal impairment		3.483	2.332	5.203	<0.001	1.635	0.952	2.807	0.750

## Discussion

Data from 1,633 individuals obtained, whose diagnoses of SARS-CoV-2 infection were established and were reviewed during the study. Evidence connecting the Ct value, which measures the viral load, with an elevated risk of death was discovered in this record-based single-center investigation.

The results notably showed that the Ct levels of SARS-CoV-2 in nasopharyngeal swabs were a strong predictor of illness prognosis and were linked to in-hospital mortality, Waudby-West et al.'s study found after controlling for all relevant clinical factors, a substantial association between the initial Ct value and death in their analysis of 1,337 individuals [10]. In a similar vein, Pujadas et al. found that in a sample of 1,145 hospitalized patients, viral load was an independent predictor of death [11].

The current study found that the frequency of hypoxia and the need for oxygen in the emergency department among patients with a low Ct value indicated with a Ct value <30 were more than those patients with a high Ct value which represented as Ct ≥30; according to research by Magleby et al., patients with a high viral load were more likely than those with a medium or low viral load to require intubation [12].

On the other hand, Salto-Alejandre et al. did not find a relationship between the number of virus copies detected in nasopharyngeal swabs and the probability of being admitted to the ICU or mortality using multivariable logistic regression [13]. Further, Argyropoulos et al.'s study showed that the viral load had an inverse correlation with the severity of COVID-19, demonstrating that the viral load was higher in patients with a less severe form of the illness and no significant association was found between viral load and admission to the ICU, length of oxygen support, and overall survival care unit (ICU) or mortality [14]. The reason for this could be due to the small sample size that was used in both studies [13,14].

In terms of laboratory markers, our study found that individuals with high viral loads showed significant elevations in markers such as CRP, D-dimer, lymphopenia, LDH, and cardiac markers, as well as low Ct levels. A systematic review that included 18 studies concluded that there was a significant relationship between increased neutrophil counts, LDH, and lower Ct levels [15].

A study by Huang et al. with 308 patients revealed a correlation between a high viral load and an increased risk of cardiac damage, elevated troponins, coagulopathy, liver and renal dysfunction, and in-hospital death [16]. Within the scope of our study, fever and cough were the most common complaints of our patients. According to Chowdhury and Oommen’s review, the most common signs and symptoms are a high temperature, followed by coughing and shortness of breath [17].

Furthermore, the present investigation showed the correlation between the socio-demographic characteristics of patients and their comorbidities with the outcome. Elderly patients had a higher mortality rate, as would be predicted given the prevalence of research citing the aging population as a risk factor. Several studies in the region revealed that old age can be an independent cause of death in COVID-19 patients [18-20].

A retrospective study conducted in Saudi Arabia on COVID-19 patients identified numerous factors that were predictive of mortality within 28 days, including advanced age and smoking [18]. Another research from AL Madina found a greater mortality risk for those aged over 65 years with elevated CRP levels, which is consistent with previous research findings [19]. According to the research conducted in Oman, the likelihood of dying while in the hospital increases with old age and chronic conditions [20]. However, a study conducted in Kuwait found that a worsening prognosis was associated with the middle-aged population [21].

The results of our study showed the predominance of the male gender over the female population in mortality but were not statistically significant. Male mortality in Italy was found to be greater than in China, according to Stadio et al., who speculated that this was because Italian men had a higher prevalence of cardiovascular disorders [22].

A large single-site electronic medical records-based cohort analysis from three hospitals under the University of Washington Medicine indicated that male sex was related to greater rates of incidence of COVID-19 as well as severe COVID-19, hospitalization, and mortality [23].

According to the current study findings, hypertension, diabetes, neurological disease, and obstructive lung disease all had a significant correlation with in-hospital mortality. A study of 32,583 patients in Mexico indicated that patients with at least one comorbid condition were much more likely to get infected and acquire serious illness [24].

The investigation revealed a significant number of fatalities, which were accompanied by an elevated level of LDH and CRP, a positive D-dimer, and a high ferritin level. According to the research findings of Barry et al.'s study, the serum lactate was shown to be a predictor of intubation and a bad outcome [25]. After examining the blood samples of 485 patients from the Wuhan area, Yan et al. found three primary laboratory indicators for the prediction of mortality. These indications were increased serum LDH, a CRP level, and lymphopenia [26].

The current research found a higher percentage of deaths among COVID-19 patients who had elevated troponin levels. The association was significant between increased troponin levels as an indicator of cardiac injury and increased probability of mortality among Rashid Hospital patients. The current study results agreed with Chehab et al.'s study on 454 hospitalized patients which showed a significant association between high troponin and death [27].

To determine the independent relationships between high rates of in-hospital mortality and advanced age, male gender, chronic illnesses, and compromised clinical and laboratory indicators, our study utilized multivariate logistic regression. Izcovich et al. evaluated 207 publications to identify risk indicators for death or illness severity that could aid in making decisions on the care of patients infected with COVID-19. Researchers identified 49 factors that independently predict mortality or severe COVID-19 illness. For instance, older people and those with pre-existing medical illnesses like chronic obstructive pulmonary disease (COPD) or cardiovascular disease had a higher risk of getting severe COVID-19 or dying from the virus [28].

The research had certain limitations. The study conclusions cannot be applied to other COVID-19 patients because it only covered individuals receiving care in a single facility. Additionally, the study can only show the association of viral load with poor outcomes but it cannot be considered a causative factor. Because of the nature of the study, it was not possible to track the outcomes of patients after they were released from the hospital.

The primary advantage of the current study, however, was that it might stimulate additional research to examine the viability of using viral load or Ct value as a tool to forecast the severity of the disease and its potential for transmission.

## Conclusions

In COVID-19 patients, there was a strong correlation between the viral load as indicated by the Ct value and death rate. Patients with COVID-19 who were older, or had concomitant conditions, and higher tissue injury markers had a greatly elevated risk of mortality. The study found that a higher mortality rate in COVID-19 individuals was independently associated with advanced age, a history of chronic disease, and aberrant clinical and laboratory findings. The data suggested that, in addition to viral load, these variables might be used as predictive and prognostic markers. Therefore, further research is advised to examine the connection between the infectivity of COVID-19, its severity, and viral load levels. Additionally, it is wise to keep an eye on and study the dynamic shift in the level of viral load throughout the illness.
